# Response Hand Differentially Affects Action Word Processing

**DOI:** 10.3389/fpsyg.2017.02223

**Published:** 2017-12-19

**Authors:** Nina Heck, Bettina Mohr

**Affiliations:** Department of Psychiatry, Campus Benjamin Franklin, Charité Universitätsmedizin Berlin, Berlin, Germany

**Keywords:** language, hemispheres, action words, motor task, visual field paradigm

## Abstract

Recent approaches in the tradition of theories of semantic and conceptual “grounding” emphasize the role of perceptual and motor knowledge in language as well as action understanding. However, the role of the two cerebral hemispheres in integrating action-motor and language processes is not clear yet. The present study looked at the influence of a simultaneous motor tapping task on word processing. In a lexical decision task, uni-manual and bi-manual hand-related, and foot-related action verbs were presented in the left and right visual half-field. A group of healthy participants performed tapping with the left hand and lexical decisions with their right hand. In a second group of participants, the reversed hand response pattern was applied. The results showed that response hand had an influence on functional lateralization of word processing when responses were executed with the non-dominant hand. Projecting words to the ipsilateral hemisphere relative to the hemisphere performing lexical decisions led to significantly decreased performance. The results showed that left hand responses led to an increased accuracy for hand-related in contrast to foot-related action verbs. The findings suggest an influence of response hand on action word processing.

## Introduction

Recent approaches on embodied cognition and theories of semantic and conceptual “grounding” emphasize the role of perceptual and motor knowledge in language as well as in action understanding (Barsalou, 2008). The recognition and understanding of observed actions and semantic action processing, in which motor systems are likely to play a key role, seem to be of major importance for communication and social interaction (Rizzolatti and Fabbri-Destro, 2010). The functional connection between the language and the motor system has been addressed in a number of studies in recent years (Pulvermüller et al., 2001, 2005; Hauk and Pulvermüller, 2004; Hauk et al., 2004; Boulenger et al., 2006; Scorolli and Borghi, 2007; Nazir et al., 2008; Borghi and Scorolli, 2009; Scorolli et al., 2009; Fargier et al., 2012a,b; Shebani and Pulvermüller, 2013; Strozyk et al., 2017). In this context, a large part of research focuses on the processing of action-related words vs. non-action words. For example, it has been shown that reading or listening to action-related words results in activation of premotor and primary motor cortex areas in addition to traditional language regions (Hauk et al., 2004; Tettamanti et al., 2005; Hauk and Pulvermüller, 2011). Further evidence for a functional link between the motor and the semantic language system comes from patients with neurodegenerative diseases affecting the motor system (Boulenger et al., 2008; York et al., 2014), autism spectrum conditions (Moseley et al., 2014, 2015) and patients with focal brain lesions in the premotor- and motor cortex (Neininger and Pulvermüller, 2001, 2003; Dreyer et al., 2015) who show a specific impairment of action word processing. In a transcranial magnetic stimulation (TMS) study with healthy participants, Pulvermüller et al. (2005) demonstrated that stimulation of the hand/arm region within the left motor cortex resulted in significantly better performance in a lexical decision task when hand-related action verbs were processed as opposed to foot-related action verbs. A similar effect was obtained for foot-related action verbs after stimulation of the foot/leg region within the left motor cortex. Buccino et al. (2005) found a modulation of motor evoked potentials of the hand muscles when participants listened to sentences containing hand-related action words while the hand/arm motor area of the left hemisphere was stimulated with single-pulse TMS. Again, a corresponding effect was found for foot-related action word sentences during stimulation of the cortical foot/leg area with TMS (Buccino et al., 2005).

Behavioral experiments using a dual task paradigm help to further investigate the mutual influence of language and motor tasks. Both, facilitating (Boulenger et al., 2006; Fargier et al., 2012a; Rodriguez et al., 2012; Strozyk et al., 2017) and inhibitory effects (Boulenger et al., 2006; Nazir et al., 2008; Shebani and Pulvermüller, 2013) have been reported when participants were performing a language task while simultaneously engaging in a motor task. An inhibitory effect of a complex hand and foot tapping task on a verbal memory task was reported (Shebani and Pulvermüller, 2013): In this study, memory performance for hand or foot action words was differentially impaired by executed hand or foot movements. In contrast, Fargier et al. (2012a) observed accelerated movements when participants simultaneously produced action-related words, but not when they produced non-action-related verbs. In a very recent study, Strozyk et al. (2017) applied a single- and dual-task condition in which participants engaged either the foot or hand with a simultaneous tapping task while performing lexical decisions on hand- or foot-related nouns. Responses for hand-related words were faster with hand reactions and foot-related words with foot responses. However, there was no differential effect of hand- and foot tapping on lexical decisions on any action word category. Boulenger et al. (2006) showed a time-dependent effect of a lexical decision task with action-related words on a grasping movement. Here, the lexical decision facilitated the movement when executed prior to a grasping action, but interfered with it, when executed after the onset of the grasping movement. Inhibitory effects of motor actions on a lexical decision task have also been reported by Nazir et al. (2008). In contrast, no specific influence of a right or left hand finger tapping task on action word processing was found in different experiments with silent and aloud reading of words (Postle et al., 2013). A study of Scorolli and Borghi (2007) showed a facilitation effect in verbal responses for mouth-related sentences, as well as for responses executed with a foot pedal for foot-related sentences relative to hand-related sentences, indicating effector specific modulation of the action-motor system. Little attention has been given so far to lateralization effects and the influence of handedness on motor-language interaction. In a functional magnetic resonance imaging (fMRI) study, Willems et al. (2010) tested right- and left-handed participants in a lexical decision task employing action words. While right-handed participants showed an activation of left hemispheric (LH) premotor cortex, left-handed participants demonstrated premotor activation in the right hemisphere (RH), indicating an influence of hand dominance on lexical processing (Willems et al., 2010). In contrast, a study by Hauk and Pulvermüller (2011) found differential motor cortex activation during language processing to be independent from hand dominance. In this study, uni-manual (actions performed with the dominant hand) and bi-manual (actions performed with both hands simultaneously) hand-related action verbs were presented to right- and left-handed participants in a silent reading task. Interestingly, the findings revealed bilateral motor cortex activation for bi-manual action words, while uni-manual action words elicited unilateral activation in areas of the motor cortex. This unilateral activation pattern was lateralized to the left hemisphere, irrespective of participants’ handedness (Hauk and Pulvermüller, 2011).

Thus, to this end, the influence of an active motor task on language processing or, more precisely, on differential processing of words belonging to different (action) semantic categories, is not fully understood. Particularly, there is a need for a more detailed examination of the influence of hemispheric differences. Therefore, we aimed to further explore lateralization effects in the interaction between the language and motor system by implementing a single and a dual-task paradigm. In the latter condition, right handed participants were asked to perform a lexical decision task while they simultaneously engaged in a complex tapping task with either the left or right hand. The differential processing of action words in the left and RHs were investigated with a divided visual field paradigm in which uni-manual and bi-manual hand-related action words, as well as foot-related action verbs were presented. Based on previous findings (Medland et al., 2002), we expected better performance in the single task condition compared to the dual task condition and better performance for words presented in the right visual field (RVF) relative to the left visual field (LVF) (Mohr et al., 1996; Knecht et al., 2000; Bourne, 2006). Most importantly, we predicted to find an effect of the tapping task on action word processing. More specifically, we predicted to find a stronger effect of hand tapping on lexical decisions for hand-related action verbs as opposed to foot-related action verbs with the strongest influence on processing uni-manual hand-related action words.

Two studies were conducted: A pilot experiment employed a stimulus rating study, in which a large corpus of word stimuli was evaluated and rated according to various psycholinguistic variables. In a dual task study with a divided visual field paradigm, we addressed the interaction of motor performance on action word processing. Written informed consent was obtained from all participants prior to their participation and all participants were reimbursed for their time. Both studies described here were approved by Charité – Universitätsmedizin Berlin Research Ethics Committee. This research was carried out in accordance with the Declaration of Helsinki for experiments involving humans.

## Stimulus Rating Study

### Methods

In order to select appropriate and well-matched uni-manual hand-related (U), bi-manual hand-related (B) and foot-related (F) action words for the lexical decision paradigm, a stimulus rating study was performed in accordance with previous studies (Pulvermüller et al., 1999; Hauk et al., 2004; Moseley et al., 2012).

#### Participants

14 right-handed, native and monolingual speakers of German (2 male, 12 female) with a mean age of 42.6 years (*SD* = 13.8) participated in an online-based stimulus rating study. None of the participants who engaged in the stimulus rating study was tested in the dual task study.

#### Procedure

Participants evaluated a selection of 144 German bi-syllabic action verbs in the infinitive form. Word length ranged from 5 to 9 letters with an average word length of 6.7 letters (*SD* = 1.1). Evaluation comprised ratings on the following variables: (1) *familiarity*, (2) *imageability*, (3) *valence*, (4) *foot-relatedness* (associations with foot movements) and (5) *hand-relatedness* (associations with hand movements), e.g., “How familiar is this word to you? Do you use or hear it frequently?” A Likert scale ranging from + 3 (e.g., highly familiar) to -3 (e.g., not familiar) was applied for stimulus evaluation. In addition, participants rated the words according to their motor association and execution with either one hand or both hands with two additional questions/variables. Variable (6) assessed the usual performance of the described action by the participants [‘How do you usually perform the described action?’] on an equivalent Likert scale (+ 3: ONLY with the LEFT HAND; 0: with BOTH HANDS simultaneously; -3: ONLY with the RIGHT HAND). Variable (7) [“Is it necessary to perform the described action with both hands or is the performance possible with one hand only?”] with three response options (1: performance of the action possible with ONE hand only; 2: BOTH hands necessary: both hands perform DIFFERENT movements; 3: BOTH hands necessary: both hands perform the SAME movements) assessed whether the actions, regardless of the usual performance, can or cannot be performed with one hand only. In addition, this variable assessed if a bi-manual action consists of a main action of the dominant hand and an assisting action of the non-dominant hand [response option 2] or consists of two identical movements of both hands [response option 3]. Furthermore, stimuli were assessed for word length and word frequency with the dlexDB database (University of Potsdam, Germany^1^).

### Results

Hand-related action verbs and foot-related action verbs were selected based on the ratings for the variables *foot-relatedness* (4) and *hand-relatedness* (5), resulting in 34 foot-related (F) and 110 hand-related action verbs. Subsequently, uni-manual hand-related verbs (U) and bi-manual hand-related verbs (B) were selected as subcategories of hand-related action verbs, based on the results for variables (6) and (7). Taking only variable (6) into consideration, ratings showed a strong tendency to rate words as describing actions that are usually performed with both hands. Out of the 110 words rated as hand-related, 90 words were rated as ‘usually performed with both hands’ and 20 words as ‘usually performed with only one hand’ concerning variable (6). Thus, we used variable (7) to identify words that describe actions that are usually performed with both hands (variable 6), but can be performed with one hand only (variable 7). These items as well as all items describing actions that are usually performed with one hand only were assigned to the uni-manual category [U]. All items that can be performed with both hands only were assigned to the bi-manual verb category [B].

From these categories, we selected 30 stimuli for each word category U, B, and F, for the final set of stimuli for the dual task study. We used Friedman Test and Wilcoxon Signed-Ranks Tests as non-parametric tests as data were not normally distributed. Friedman test revealed no significant differences (all *p*-values > 0.05) between the word categories with regards to the variables *familiarity, imageability, valence, length* and *frequency*.

Median, means, and standard deviants for psycholinguistic variables (1–3) as well as length and word frequency are displayed in **Table 1**.

**Table 1:** Median (MD), Means (M), and Standard Deviations (SD) for psycholinguistic variables for the word categories derived from the pilot study: uni-manual hand-related (U), bi-manual hand-related (B), and foot-related (F) action words with 30 items per category.

Psycholinguistic variable	Uni-manual (U) words	Bi-Manual (B) words	Foot (F) words
Familiarity (1)	*MD* = 2.54	*MD* = 2.04	*MD* = 2.21
	*M* = 2.25	*M* = 2.03	*M* = 2.08
	*SD* = 0.67	*SD* = 0.78	*SD* = 0.75
Imageability (2)	*MD* = 2.07	*MD* = 1.96	*MD* = 1.43
	*M* = 1.84	*M* = 1.85	*M* = 1.58
	*SD* = 0.85	*SD* = 0.96	*SD* = 0.81
Valence (3)	*MD* = 0.43	*MD* = 0.57	*MD* = 0.25
	*M* = 0.04	*M* = 0.62	*M* = 0.19
	*SD* = 1.06	*SD* = 0.46	*SD* = 1.16
Word frequency	*MD* = 167	*MD* = 122	*MD* = 144
	*M* = 1175	*M* = 294	*M* = 992
	*SD* = 3494	*SD* = 518	*SD* = 2060
Word length (in letters)	*MD* = 7	*MD* = 6	*MD* = 7
	*M* = 6.83	*M* = 6.4	*M* = 6.67
	*SD* = 1.21	*SD* = 1.00	*SD* = 0.92

After careful selection of 90 stimuli, 90 pronounceable, orthographically regular pseudowords were generated which were not homophonic to real German words. Pseudowords were matched for item length.

## Dual Task Study

### Methods

#### Participants

Thirty-one healthy participants took part in the experiment and were randomly divided into Group R (right hand tapping) and Group L (left hand tapping). The mean overall accuracy (*M, SD*) was comparable with other studies with a lexical decision task (Mohr et al., 1996). Nevertheless, we excluded all participants with a mean accuracy of less than 65% for all stimuli (words and pseudowords) in the lexical decision task. Thus, the results of 26 participants [a total of 13 participants (3 male and 10 females) in each group] entered the final analysis. All participants were monolingual, German native speakers. The mean age of the 26 participants was 25.9 years [Group R: 26.7 (*SD* = 6.6) and Group L: 25.2 (*SD* = 4.5)]. All participants had normal or corrected-to-normal vision and were right handed as assessed with the 10-item version of the Edinburgh Handedness Questionnaire (Oldfield, 1971) with a mean laterality index of 90.4 (*SD* = 13.4) [Group R: *M* = 94.6, *MD* = 100 (*SD* = 9.7) and Group L: *M* = 86.2, *MD* = 90 (*SD* = 15.6)]. No significant difference was found between the mean laterality index of the two groups [*U* = -1.802; *p* > 0.05].

#### Procedure

**FIGURE 1:**
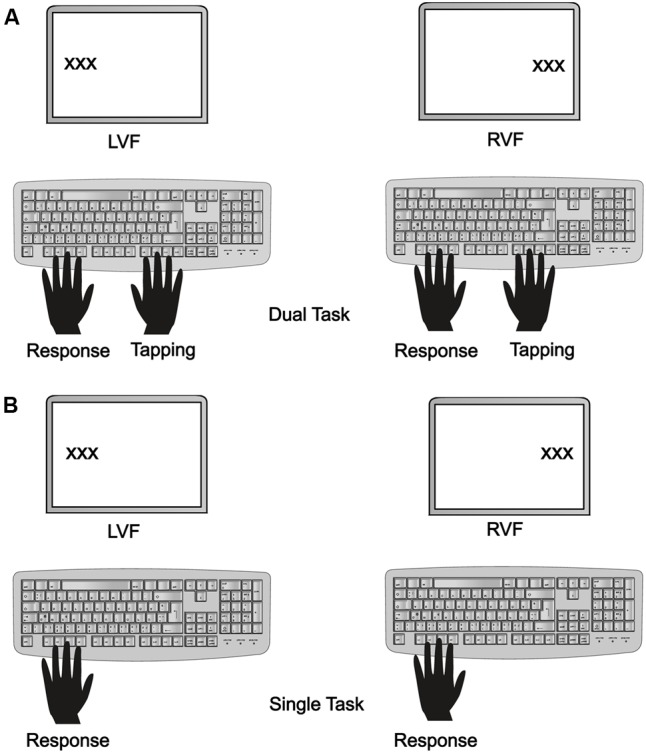
Experimental setup for stimulus presentation in all four conditions including presentation in left visual field (LVF) (Left) and right visual field (RVF) (Right) in the dual task **(A)** and the single task **(B)** condition, displayed for Group R who performed the tapping with the right hand and lexical decisions with the left hand.

The experiment consisted of a single task and a dual task condition. In the single task condition, participants had to perform a lexical decision task without an additional motor task. In the dual task condition, they performed lexical decisions while simultaneously engaging in tapping. Group R performed the tapping task with the right hand and executed lexical decisions with their left hand. Group L performed the tapping task with the left hand and responded with their right hand. Before the start of the main experiment, participants practiced the tapping sequence as well as the lexical decision task with a different set of word stimuli. During the experiment, each stimulus was repeated four times and was presented in the LVF in the single task condition (a), in the LVF in the dual task condition (b), as well as in the RVF in the single task condition (c) and in the RVF in the dual task condition (d), respectively (see **Figures 1A,B** for details). The experiment started either with a single task sequence or a dual task sequence.

The experiment took place in a quiet room. Participants’ head was placed on a chin rest with a forehead restraint bar to minimize head movements. The viewing distance was 60 cm. Each stimulus was presented with an inner visual angle of 0.4° and an outer visual angle of 2.9° to the left or to the right of the central fixation point in accordance with the recommendation of Bourne (2006). The start of the experiment was initiated by the participant. The study was divided in four parts (two single task and two dual task sections) including 180 stimuli (90 words and 90 pseudowords) each. Each experimental part was further subdivided in two subsections including 90 items each. All subsections were separated by breaks and participants were encouraged to take breaks and determine the duration of the breaks before resuming the experiment. At the beginning of the experiment, as well as after each break, an instruction, indicating the condition (no tapping or tapping) was displayed for 3 s. A green display with the instruction ‘TAPPING until the next break’ announced the subsequent dual task condition while a red display with the instruction ‘NO TAPPING until the next break’ announced a subsequent single task condition.

##### Lexical decision task

**FIGURE 2:**
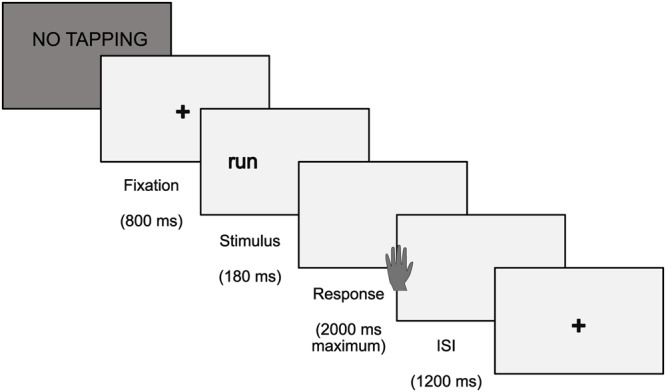
Procedure and timing of the lexical decision task displayed exemplarily for stimulus presentation in the LVF.

**FIGURE 3:**
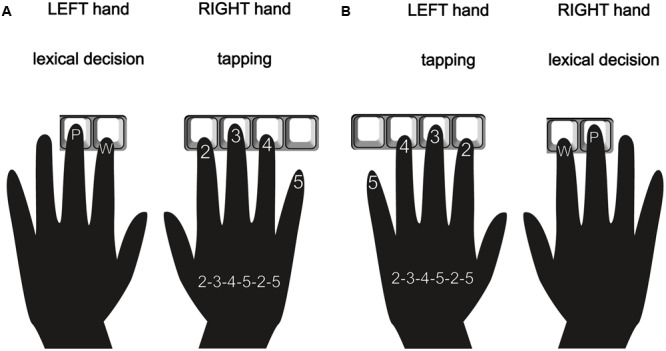
Finger tapping sequence and lexical decision response demonstrated for Group R **(A)** who performed the tapping task with the right hand and responded to words with their left hand as well as for Group L **(B)** who performed the tapping task with the left hand and responded to words with their right hand.

In each trial, a fixation cross was presented in the center of the screen for 800 ms. The fixation cross was then replaced by a stimulus. A divided visual field paradigm was chosen in which stimuli were presented in a randomized fashion either in the LVF or the RVF for 180 ms. The stimulus presentation was followed by a blank screen for a maximum of 2000 ms. During this time interval, participants had to decide whether a letter string was a real word or a pseudoword by pressing one out of two answer keys on a computer keyboard. Participants were instructed to respond as accurately and as quickly as possible. The inter stimulus interval (ISI) lasted for 1200 ms (see **Figure 2** for details). Stimuli were presented in four different, pseudo-randomized trial lists which were re-used and counterbalanced between study participants. Participants responded with the index (words) and middle finger (pseudowords) of their left hand (Group R) or with the respective fingers of their right hand (Group L) (see **Figures 3A,B**).

##### Tapping task

In the dual task condition, participants were required to perform a one-handed tapping sequence with four fingers [index (2), middle (3), ring (4) and little finger (5)] in a specific sequence: 2-3-4-5-2-5 finger (see **Figures 3A,B**). Participants were instructed to start the tapping immediately after appearance of the display of the dual task condition. The participants were instructed to perform the tapping continuously throughout the dual task conditions in their preferred speed. Furthermore, participants were instructed that no specific rhythm was required and only the correct sequence of keystrokes was important. In addition, participants were told to restart the tapping sequence at any position of the sequence in case of interruption.

### Results

Accuracies, latencies and tapping frequencies (inter-tap-intervals) for all experimental conditions were analyzed using SPSS statistical software. Repeated measures analyses of variance (ANOVAs) were performed with the between subject factor *Group* (Group L: left hand tapping vs. Group R: right hand tapping) and the within-subject factors *Visual Field* (LVF vs. RVF), *Task* (single task/no tapping vs. dual task/tapping) and *Lexicality* [words vs. pseudowords; see section Lexicality (Words vs. Pseudowords) Effects]. As a next step, only word responses were analyzed and the factor *Word Category* (three levels: uni-manual hand-related action verbs [U]; bi-manual hand-related action verbs [B]; foot-related action verbs [F]) replaced the factor *Lexicality* (see section Word Category Differences). Statistically significant interactions were further analyzed with *post hoc t*-tests with Holm-Bonferroni correction. Descriptive statistics, including means (M), medians (MD) and standard deviants (SD) for each group and condition regarding lexicality are provided in **Table 2**. Descriptive statistics regarding word categories are provided in **Table 3**.

**Table 2:** Dual task study: Means (M), Median (MD), and Standard Deviations (SD) for accuracies, latencies and tapping frequencies (inter-tap-intervals) obtained for words (W) and pseudowords (P) in all experimental conditions.

			Single Task (no tapping)		Dual Task (tapping)	
			W	P	W	P
Group L	LVF	Accuracies	*M* = 0.76	*M* = 0.86	*M* = 0.70	*M* = 0.78
			*MD* = 0.77	*MD* = 0.87	*MD* = 0.71	*MD* = 0.82
			*SD* = 0.15	*SD* = 0.06	*SD* = 0.15	*SD* = 0.15
		Latencies	*M* = 816	*M* = 863	*M* = 905	*M* = 949
			*MD* = 823	*MD* = 935	*MD* = 850	*MD* = 892
			*SD* = 84	*SD* = 88	*SD* = 121	*SD* = 114
		Tapping frequency			*M* = 458	*M* = 461
					*MD* = 468	*MD* = 476
					*SD* = 90	*SD* = 99
	RVF	Accuracies	*M* = 0.84	*M* = 0.87	*M* = 0.78	*M* = 0.80
			*MD* = 0.86	*MD* = 0.87	*MD* = 0.85	*MD* = 0.85
			*SD* = 0.09	*SD* = 0.09	*SD* = 0.17	*SD* = 0.14
		Latencies	*M* = 776	*M* = 844	*M* = 872	*M* = 929
			*MD* = 754	*MD* = 831	*MD* = 860	*MD* = 906
			*SD* = 80	*SD* = 84	*SD* = 99	*SD* = 85
		Tapping frequency			*M* = 458	*M* = 461
					*MD* = 478	*MD* = 474
					*SD* = 97	*SD* = 98
Group R	LVF	Accuracies	*M* = 0.67	*M* = 0.87	*M* = 0.62	*M* = 0.83
			*MD* = 0.68	*MD* = 0.9	*MD* = 0.63	*MD* = 0.83
			*SD* = 0.14	*SD* = 0.1	*SD* = 0.2	*SD* = 0.12
		Latencies	*M* = 859	*M* = 890	*M* = 888	*M* = 878
			*MD* = 857	*MD* = 868	*MD* = 881	*MD* = 852
			*SD* = 100	*SD* = 144	*SD* = 129	*SD* = 163
		Tapping frequency			*M* = 478	*M* = 483
					*MD* = 492	*MD* = 494
					*SD* = 51	*SD* = 43
	RVF	Accuracies	*M* = 0.88	*M* = 0.85	*M* = 0.88	*M* = 0.81
			*MD* = 0.9	*MD* = 0.85	*MD* = 0.91	*MD* = 0.81
			*SD* = 0.05	*SD* = 0.07	*SD* = 0.08	*SD* = 0.1
		Latencies	*M* = 756	*M* = 872	*M* = 805	*M* = 884
			*MD* = 749	*MD* = 848	*MD* = 832	*MD* = 861
			*SD* = 115	*SD* = 136	*SD* = 141	*SD* = 130
		Tapping frequency			*M* = 470	*M* = 483
					*MD* = 474	*MD* = 496
					*SD* = 43	*SD* = 45

**Table 3:** Dual task study: Means (M), Medians (MD), and Standard Deviations (SD) for accuracies, latencies and tapping frequencies (inter-tap-intervals) obtained for uni-manual hand-related (U), bi-manual hand-related (B) and foot-related (F) action verbs in all experimental conditions.

			Single Task (no tapping)			Dual Task (tapping)		
			U	B	F	U	B	F
Group L	LVF	Accuracies	*M* = 0.78	*M* = 0.75	*M* = 0.75	*M* = 0.67	*M* = 0.72	*M* = 0.7
			*MD* = 0.88	*MD* = 0.77	*MD* = 0.77	*MD* = 0.62	*MD* = 0.69	*MD* = 0.69
			*SD* = 0.18	*SD* = 0.17	*SD* = 0.13	*SD* = 0.16	*SD* = 0.18	*SD* = 0.16
		Latencies	*M* = 833	*M* = 801	*M* = 812	*M* = 920	*M* = 907	*M* = 890
			*MD* = 818	*MD* = 789	*MD* = 814	*MD* = 891	*MD* = 906	*MD* = 860
			*SD* = 102	*SD* = 81	*SD* = 82	*SD* = 130	*SD* = 123	*SD* = 137
		Tapping Frequency				*M* = 490	*M* = 487	*M* = 499
						*MD* = 501	*MD* = 499	*MD* = 511
						*SD* = 90	*SD* = 75	*SD* = 106
	RVF	Accuracies	*M* = 0.84	*M* = 0.8	*M* = 0.88	*M* = 0.81	*M* = 0.79	*M* = 0.76
			*MD* = 0.85	*MD* = 0.85	*MD* = 0.92	*MD* = 0.88	*MD* = 0.81	*MD* = 81
			*SD* = 0.14	*SD* = 0.13	*SD* = 0.09	*SD* = 0.18	*SD* = 0.13	*SD* = 0.2
		Latencies	*M* = 780	*M* = 774	*M* = 776	*M* = 861	*M* = 874	*M* = 882
			*MD* = 791	*MD* = 780	*MD* = 759	*MD* = 849	*MD* = 837	*MD* = 858
			*SD* = 95	*SD* = 52	*SD* = 102	*SD* = 98	*SD* = 95	*SD* = 131
		Tapping Frequency				*M* = 484	*M* = 480	*M* = 488
						*MD* = 493	*MD* = 488	*MD* = 509
						*SD* = 95	*SD* = 91	*SD* = 98
Group R	LVF	Accuracies	*M* = 0.7	*M* = 0.67	*M* = 0.65	*M* = 0.65	*M* = 0.64	*M* = 0.59
			*MD* = 0.7	*MD* = 0.73	*MD* = 0.65	*MD* = 0.62	*MD* = 0.65	*MD* = 0.62
			*SD* = 0.15	*SD* = 0.17	*SD* = 0.17	*SD* = 0.2	*SD* = 0.21	*SD* = 0.21
		Latencies	*M* = 848	*M* = 873	*M* = 854	*M* = 879	*M* = 885	*M* = 906
			*MD* = 837	*MD* = 856	*MD* = 844	*MD* = 896	*MD* = 903	*MD* = 904
			*SD* = 82	*SD* = 115	*SD* = 111	*SD* = 144	*SD* = 145	*SD* = 139
		Tapping Frequency				*M* = 459	*M* = 467	*M* = 431
						*MD* = 479	*MD* = 494	*MD* = 486
						*SD* = 82	*SD* = 74	*SD* = 145
	RVF	Accuracies	*M* = 0.88	*M* = 0.91	*M* = 0.86	*M* = 0.88	*M* = 0.89	*M* = 0.86
			*MD* = 0.88	*MD* = 0.92	*MD* = 0.88	*MD* = 0.92	*MD* = 0.92	*MD* = 0.88
			*SD* = 0.08	*SD* = 0.07	*SD* = 0.08	*SD* = 0.09	*SD* = 0.09	*SD* = 0.1
		Latencies	*M* = 739	*M* = 770	*M* = 758	*M* = 781	*M* = 817	*M* = 816
			*MD* = 730	*MD* = 769	*MD* = 735	*MD* = 782	*MD* = 832	*MD* = 856
			*SD* = 113	*SD* = 114	*SD* = 125	*SD* = 126	*SD* = 162	*SD* = 152
		Tapping Frequency				*M* = 456	*M* = 470	*M* = 455
						*MD* = 475	*MD* = 483	*MD* = 465
						*SD* = 67	*SD* = 74	*SD* = 58

#### Lexicality (Words vs. Pseudowords) Effects

##### Accuracies

**FIGURE 4:**
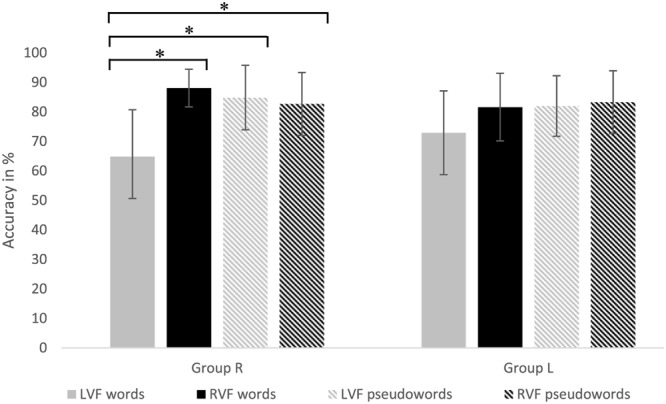
Accuracies to words and pseudowords presented either in the RVF or LVF for Group R (Left) and Group L (Right) are presented. Significant differences are marked with ^∗^.

Data showed a main effect of *Task* [*F*(1,24) = 12.84; *p* < 0.01, $\eta_{p}^{2}$ = 0.35] with an overall higher accuracy in the single task condition as opposed to the dual task condition. Furthermore, data showed a main effect of *Lexicality* [*F*(1,24) = 6.86; *p* < 0.05, $\eta_{p}^{2}$ = 0.22] and *Visual Field* [*F*(1,24) = 18.02, *p* < 0.0001, $\eta_{p}^{2}$ = 0.43], a significant two-way interaction *Visual Field* × *Lexicality* [*F*(1,24) = 29.17, *p* < 0.0001, $\eta_{p}^{2}$ = 0.55] and a three-way interaction *Visual Field* × *Lexicality* × *Group* [*F*(1,24) = 9.32, *p* < 0.01, $\eta_{p}^{2}$ = 0.28] (see **Figure 4**). *Post hoc* tests of the interaction *Visual Field* × *Lexicality* × *Group* revealed a significant difference between words in the LVF [*M* = 0.65, *SD* = 0.16] and words in the RVF [*M* = 0.88, *SD* = 0.06] (right visual field advantage, RVFA) in Group R [*t*(12) = 4.01, *p* < 0.01, *d* = 1.45]. In contrast, no significant differences between words and pseudowords presented in LVF and RVF were found for Group L. Furthermore, significant lower performance for words in the LVF as opposed to pseudowords in the RVF [*M* = 0.83, *SD* = 0.07] and LVF [*M* = 0.85, *SD* = 0.1] was found for Group R. This three-way interaction is displayed in **Figure 4**.

##### Latencies

**FIGURE 5:**
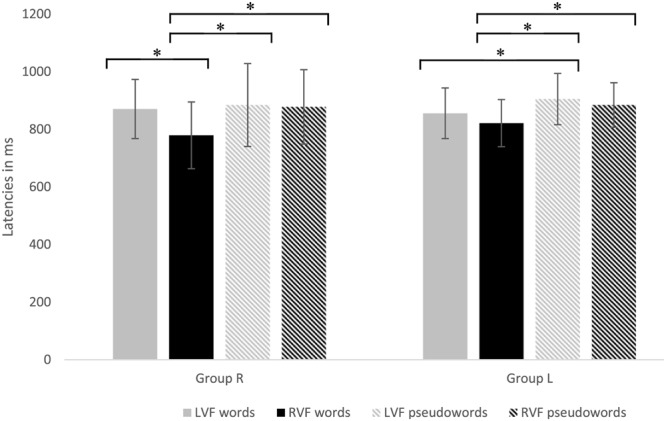
Latencies to words and pseudowords presented either in the RVF or LVF for Group R (Left) and Group L (Right) are presented. Significant differences are marked with ^∗^.

Data showed a main effect of *Visual Field* [*F*(1,24) = 45.14, *p* < 0.0001, $\eta_{p}^{2}$ = 0.65] and *Task* [*F*(1,24) = 14.26, *p* < 01, $\eta_{p}^{2}$ = 0.37] as well as a main effect of *Lexicality* [*F*(1,24) = 32.11, *p* < 0.0001, $\eta_{p}^{2}$ = 0.57]. In addition, data revealed significant two-way interactions of the factors *Group* × *Task* [*F*(1,24) = 5.77, *p* < 0.05, $\eta_{p}^{2}$ = 0.19] and *Visual Field* × *Lexicality* [*F*(1,24) = 19.37, *p* < 0.0001, $\eta_{p}^{2}$ = 0.45], as well as a three-way interaction of *Group* × *Visual Field* × *Lexicality* [*F*(1,24) = 8.97, *p* < 0.01, $\eta_{p}^{2}$ = 0.27]. *Post hoc* analyses of the interaction *Group* × *Task* showed significant shorter latencies for the single task conditions [*M* = 824, *SD* = 80] compared to the dual task conditions [*M* = 910, *SD* = 97] for group L [*t*(12) = 4.22, *p* < 0.01, *d* = 0.98] only. *Post hoc* tests for the three-way interaction *Group* × *Visual Field* × *Lexicality* showed significantly shorter latencies for words in the RVF relative to words in the LVF for Group R [*t*(12) = 5.64, *p* < 0.0001, *d* = 0.79], but not for Group L. Further significant results for words in one visual fields compared to pseudowords in the same or the contralateral visual field in both groups reflect the overall better performance (shorter latencies) for words [*M* = 826, *SD* = 97] than pseudowords [*M* = 888, *SD* = 109]. This three-way interaction is displayed in **Figure 5**.

##### Tapping frequencies

Analysis of tapping frequencies (inter-tap-intervals) did not reveal any significant main effects or interactions.

#### Word Category Differences

##### Accuracies

**FIGURE 6:**
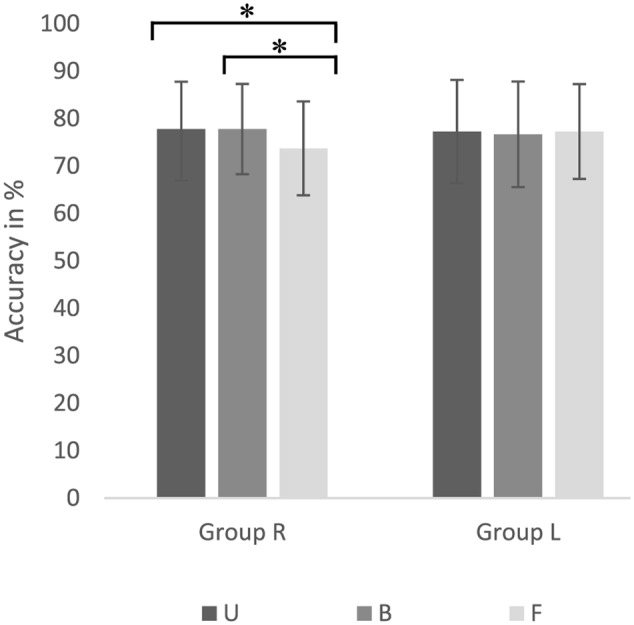
Accuracies for uni-manual hand-related [U], bi-manual hand-related [B] and foot-related action verbs are presented for Group R (Left) and Group L (Right). Significant differences are marked with ^∗^.

Accuracy analysis revealed a two-way interaction *Group* × *Word Category* [*F*(2,23) = 3.55, *p* < 0.05, $\eta_{p}^{2}$ = 0.24]. *Post hoc* analysis showed significant differences between the different word categories for Group R only. A significant better performance [*t*(12) = 3.77, *p* < 0.01, *d* = 0.4] for word category bi-manual hand-related (B) action verbs [*M* = 0.78, *SD* = 0.1] than word category foot-related (F) action verbs [*M* = 0.74, *SD* = 0.1], as well as better performance [*t*(12) = 3.19, *p* < 0.01, *d* = 0.4] for word category uni- manual hand-related (U) [*M* = 0.78, *SD* = 0.1] than F was found. No significant differences were found between the hand-related subcategories U and B [*t*(12) = 0.00, *p* > 0.05]. 85% (11) of the participants showed a facilitation effect with higher accuracies for hand-related than foot-related action verbs, 7.5% (1) of participants showed an interference effect with higher accuracies for foot-related than hand-related action verbs and 7.5% (1) of participants showed no difference between hand- and foot-related action verbs. Furthermore, no difference between word categories were found for Group L. This two-way interaction is displayed in **Figure 6**.

##### Latencies and tapping frequencies

Neither latency analysis nor analysis of tapping frequencies (inter-tap-intervals) revealed any significant main effects or interactions for the different word categories.

## Discussion

### Dual Task Decrement

As hypothesized, we found an overall better performance (accuracies) in the single task condition, where no tapping was required as opposed to the dual task condition, where a complex tapping task had to be executed while performing lexical decisions. This decrease of performance is a robust effect, referred to as “dual task decrement” (see Medland et al., 2002, for review). It is assumed to result from increased information processing demands in the dual task condition and leads to longer processing times and/or increased error rates. For latencies, we found a dual task decrement only in Group L who performed lexical decisions with their dominant right hand and the complex tapping task with their left hand. Thus, the dual task decrement for latencies in Group L may reflect higher task demands in performing the complex tapping task with the non-dominant hand, resulting in slowing of motor hand responses in the dual task condition.

### Right Visual Field Advantage (RVFA)

The significant three-way interaction of the factors *Group* × *Lexicality* × *Visual Field* in latencies and accuracies demonstrated that a RVFA for words occurred only in Group R. The RVFA, a better performance for words presented in the RVF in contrast to the LVF, has been demonstrated by many studies before and indicates left-hemispheric superiority for processing words (Hernandez et al., 1992; Mohr et al., 1994). Nevertheless, the RVFA was found only for group R, although all participants were right handed. As the RVFA not only occurred in the dual task condition, but also in the single task condition, it cannot be attributed to the influence of tapping. Instead, lexical decisions seem to have been strongly influenced by (left) hand motor responses: This is confirmed by the fact that performance for words presented in the LVF (directly projecting to the non-dominant RH) was worse when participants responded with their left hand than with their right hand. This finding suggests that when lexical information and motor responses are primarily processed by the same hemisphere, language performance decreases. This finding suggests that an inhibitory interference effect might have occurred between learnt word representations and the motor system, which possibly share overlapping neuronal networks (Hauk and Pulvermüller, 2011). Wendt et al. (2007) found worse performance for right hand response to RVF presentation and for left hand response to LVF presentation in right-handers. This interference effect was explained by ongoing inhibitory processes when the same hemisphere is primarily in charge of processing stimuli and controlling the response hand (Wendt et al., 2007). In contrast, Iacoboni and Zaidel (1996) found a facilitation effect, a better performance for hand responses ipsilateral to the visual field presentation (Iacoboni and Zaidel, 1996). Our results for Group R are consistent with the findings of Wendt et al. (2007), showing decreased performance, for (left) hand word responses in the LVF.

### Word Category Differences

Better performance (accuracies) for hand-related as opposed to foot-related action words for Group R, but not for Group L, was found. This effect occurred during single and dual task conditions. As tapping is performed throughout the entire dual task condition, continuous activation of the contralateral motor cortex during dual task conditions can be assumed. In contrast, the hand movement for the response only occurred during the execution of lexical decision responses, resulting in an activation of the contralateral hemisphere during the response only.

We expected to find a specific effect of tapping on action verb processing, however, we could not confirm our hypothesis. Interestingly, this finding is in line with the results of the study by Strozyk et al. (2017) who did not report a body part specific effect of a simultaneous hand- or foot-tapping task. In our study, the movement of the responding hand led to a specific effect at least in one group, thus, we assume that the continuous activation of the motor cortex may have diminished interaction effects while recurring activation, due to responses, influenced action verb processing. In line with previous studies (Boulenger et al., 2006; Fargier et al., 2012a), we here found a specific effect for hand-related action word processing in an experiment with simultaneous manual movements. This effect occurred in Group R who performed responses with the left hand, most likely resulting in activation of the right motor cortex. Hence, in Group R, action verb processing involved the language-dominant left hemisphere, while the lexical decision motor response was controlled by the contralateral RH. Thus, the lack of concurrence of the language and motor tasks within the same hemisphere might have led to better performance in Group R only. In contrast, in Group L, interference effects between the lexical and motor task within the same hemisphere might have taken place.

Several studies indicate a somatotopic activation of the motor cortex in action word processing (Hauk et al., 2004; Grisoni et al., 2016), thus, it could be assumed that our results reflect overlapping and body-part specific neuronal resources in processing action verbs and hand movements. In contrast to Shebani and Pulvermüller (2013), who found an inhibitory effect of hand-, or foot-tapping on recalling hand-related or foot-related action verbs, we here find a facilitating effect for hand-related words. As this effect occurred during single and dual task conditions, it might be attributed to the response hand, but less likely to the tapping movement of the contralateral hand.

We did not find any differences between uni-manual and bi-manual hand-related action verbs, as expected, based on previous MRI data (Hauk and Pulvermüller, 2011). While differential processing for uni-manual hand-related action verbs (left lateralized motor cortex activation) in contrast to bi-manual hand-related action verbs (bilateral motor cortex activation) were reported in this previous study, our behavioral results do not confirm this finding. The following points may need to be taken into consideration: First, data concerning hand-related action verb processing in fMRI studies are not consistent (see section Introduction). Second, a lack of word category differences might be attributed to stimulus properties. The pilot study showed that participants rated a high percentage of hand-related action verbs to be associated with actions they usually perform with both hands simultaneously; and only few words were associated with actions usually performed with just one hand (variable/question 6). Following the stimulus rating study by Hauk and Pulvermüller (2011), variable 6 was implemented, to distinguish between uni-manual and bi-manual hand-related words. Nevertheless, while Hauk and Pulvermüller (2011) created two distinct categories with this question, we were not able to create a category of uni-manual action words based on this variable only (see section Stimulus Rating Study). As this question refers to the described action rather than to semantic criteria, different languages (English vs. German) can hardly explain these different results in the rating studies. The difficulty in creating a strong category of purely uni-manual hand-related action words as well as the procedure for categorization in our pilot study might explain the non-significant findings with regards to processing of the subcategories of hand-related action verbs.

### Limitations

The overall accuracy of all participants was 0.73 (*SD* = 0.14) and thus is comparable to other studies with a lexical decision design (Mohr et al., 1996). However, our sample size might not have been large enough to reveal significant statistical differences in sub-analyses focusing on different action word categories in single- and dual-task conditions. Therefore, further studies with larger numbers of participants are desirable to replicate these findings. Less complex designs, focusing on only one relevant experimental factor (e.g., visual field) might be preferable to increase the statistical power for sub-analyses on word-category differences.

Another potential problem in the present experimental setup could be related to the nature of the motor task in the dual task condition, compared to those employed in previous studies (Nazir et al., 2008; Fargier et al., 2012a; Postle et al., 2013; Shebani and Pulvermüller, 2013; Strozyk et al., 2017). While in some experiments, the motor task was performed earlier than the language task (Shebani and Pulvermüller, 2013), tapping and lexical decisions were performed simultaneously in the dual task condition of the present study. Furthermore, the tapping task itself was challenging. While other studies employed a simple one-finger tapping (Postle et al., 2013) or a simple four step sequence (Strozyk et al., 2017), our participants performed a complex one-hand tapping task. As very complex dual task paradigms can diminish experimental effects (Medland et al., 2002), the lack of word category effects in our study could be attributed to this factor.

## Conclusion and Outlook

Our results show that the response hand, but not tapping, has a facilitating influence on hemispheric processing of action words, more specifically on hand-related, but not foot-related action verbs. Thus, when action word responses involve activation of hand areas within the motor cortex, language performance might be better. This may be attributed to facilitating effects within neuronal language-motor networks. Furthermore, our results indicate that when action words and motor responses are primarily processed by the same hemisphere (RH), language performance seems to decrease, which might be related to intra- hemispheric interference effects. Further research is necessary to address the interaction between language and action-motor processing.

## Author Contributions

Study concept and design: NH and BM. Programming, data collection, and statistical analysis: NH. Data interpretation: NH and BM. Manuscript wrote-up: NH and BM.

## Conflict of Interest Statement

The authors declare that the research was conducted in the absence of any commercial or financial relationships that could be construed as a potential conflict of interest.

## Footnotes

1 http://www.dlexdb.de/
